# Study on LT Accuracy Improvement by Calibration Based on Network Measurements

**DOI:** 10.3390/s21227479

**Published:** 2021-11-10

**Authors:** Jesús Velázquez, Javier Conte, Ana Cristina Majarena, Jorge Santolaria

**Affiliations:** 1 Design and Manufacturing Engineering Department, University of Zaragoza, 50018 Zaragoza, Spain; jesusve@unizar.es (J.V.); jconte@unzar.es (J.C.); jsmazo@unizar.es (J.S.); 2 Instituto de Investigación en Ingeniería de Aragón (I3A), 50018 Zaragoza, Spain

**Keywords:** calibration, laser tracker, network measurement, accuracy, working environment conditions

## Abstract

Laser trackers (LT) are widely used to calibrate other machines. Nevertheless, very little is known about calibrating an LT. There are some standards that allow us to evaluate the LT performance. However, they require specialized equipment. A calibration procedure to improve the LT accuracy in an easy and fast way is presented in this paper. This method is based on network measurements where a set of reflectors were measured from different LT positions in a working environment. The methodology proposed deal with the lack of nominal data of the reflector mesh. A measurement scenario was defined, based on error parameter dependence on distances and angles, thus, obtaining those positions more sensitive to errors. The influence of the incidence angle of the laser beam on the reflector was characterized, revealing that its contribution to the LT measurement error can be up to 13 µm. Error kinematic parameters were identified to provide the optimum value of an objective function, where the reflector mesh nominal data were unknown. The calibration procedure was validated with nominal data, by measuring a set of reflectors located on a coordinate measuring machine. The findings of this study suggested that the LT accuracy can be improved up to 25%. Moreover, the method can be carried out by the LT user without requiring specialized equipment.

## 1. Introduction

Advances in industrial robots for manufacturing systems have allowed increases in manufacturing and a more sustainable production. An appropriate measurement system allows us to determine performance of industrial machines. In fact, the study of accuracy improvement has become an important aspect of measurement systems.

Laser trackers (LTs) are non-contact measurement systems. Their applicability is increasing considerably calibrating other instruments and machines. Due to their advantages in terms of wide measurement range, accuracy, reliability, portability, high speed in data acquisition, [1], high sampling rate and automatic target tracking [2], these instruments are widely used in verification of large-scale pieces.

Many recent studies have focused on calibrating different mechanisms using an LT as a measuring instrument. In Gaudreault et al. [3], an industrial robot arm was calibrated by means of self-calibration. An LT was used to validate the calibration performance. An industrial robot was calibrated by an LT using an error model with 36 parameters and a full pose measurement method [4]. A five-axis machine tool was calibrated using an LT. Three spherically mounted reflectors were attached to the worktable, thus constructing a workpiece frame. Reflector spatial coordinates were given by the LT. The tool frame pose relative to the workpiece was then calculated [5]. An LT was used to validate a multilateration tracking system based on absolute distance measurement [6]. Robot reference positions were measured by this instrument to compare them with robot outputs [7]. The gravitational telescope deformation was modelled using an LT, a laser scanner, and an electronic distance meter [8]. In Theissen et al. [9], serial articulated robots were calibrated using an LT and a loaded double ball bar. In Zhao et al. [10], the authors developed a method that trained a neural network to estimate nonlinear residual errors.

LTs are also used to perform measurements in industrial processes. For instance, in Zhang et al. [11], the authors fabricated composite laminates by rapid hot press process. To do this, prepreg was used. Residual strains and deformation of laminates were then measured. In Tordal et al. [12], this instrument was used to obtain the relative motion (position and orientation), using an extended Kalman filter, between two ships. They were also used in medical applications such as the alignment telescope of a heavy ion medical machine, combined with a laser tracker by means of network [13].

Although LTs are widely used in many applications to perform measurements and to calibrate other instruments, there are few researchers focusing on calibrating an LT.

LTs can present errors such as non-linearities, offsets, misalignments, or eccentricities. This type of errors affects the measured coordinates, and usually, the end-user can-not know if the LT is working properly [14]. Loser and Kyle presented a geometric error model for a laser tracker having a beam steering mirror [15]. Then, this model was extended for trackers without a beam steering mirror by Muralikrishnan et al. [16]. The ASME B89.4.19 standard defines some ranging test to evaluate the LT performance that required specialized equipment.

The performance evaluation of laser trackers was analyzed in Wang et al. [17]. In Ulrich et al. [18], kinematic properties of an LT were improved by means of a measurement model that was self-regulated. The tool center point of an industrial robot was tracked with the LT. Both LT and photogrammetry were used in order to measure common points improving its measurement accuracy [19].

Calibration procedure allows us to improve model accuracy. It consists of several phases: determination of the kinematic model, data acquisition, geometric parameter identification, identification of the error sources, and finally, implementation of correction models to establish corrections in measurement results. Thus, the effects of the influence of variables in the final measurement can be quantified.

The kinematic model establishes mathematical relations and obtains non-linear equations by relating the joint variables with the position and orientation of the end-effector [20]. This system can be developed by the Denavit–Hartenberg (D–H) method [21]. In Diao et al. [22], an additional rotation was introduced to solve some of its limitations.

The measurement scenario may be taken into account. Configuration station of this instrument was studied in Zhu et al. [23]. Large-scale measurement conditions and non-uniform temperature field were analyzed.

Network measurements consist of measuring a set of targets, placed in fixed locations, by the instrument located in different positions. The nominal target position is unknown. This method does not require a long time, does not need specialized instruments and provides multi-position tests and the angular rate [24]. In Hughes et al. [25], a method based on network for parameter estimation was presented. Network methods have been used to calibrate different systems. A terrestrial laser scanner was calibrated in Lichti et al. [26], using a network of signalized points. The proposed method reduced the magnitude of residuals. In Xiong et al. [27], a new method to optimize network layouts was presented in order to improve the measurement accuracy. This work showed that the element position presented an important contribution to the accuracy and to the measurement range.

This work aims to improve the LT accuracy by means of calibration based on network measurements, for an LT having the beam source in the rotating head. The measurement scenario was designed in a working environment in order to consider those points which are more sensitive to errors, thus obtaining a reflector mesh setup. A reflector gauge was designed and measured by a CMM (Coordinate Measuring Machine) to address the lack of nominal data for the optimization process. The reflector mesh and the reflector gauge were then measured by an LT located in different positions in the workshop. The kinematic parameters that minimize the difference between the distances measured of every pair of reflectors from all LT positions were obtained. These parameters were used to correct the LT measurements using two different criteria. Finally, the LT behavior was evaluated, with the kinematic parameters obtained previously, in other positions different from those used in the optimization process. Moreover, the influence of the incidence angle of the laser beam on the reflector was analyzed to show the contribution of this component to the LT measurement error. The calibration performed does not require specialized equipment, is fast and easy, allowing an LT end-user to carry out the calibration.

## 2. Materials and Methods

### 2.1. Materials

#### 2.1.1. Laser Tracker

An LT is a measurement instrument that provides measurements of large objects in industrial environments in a fast way and in a range of more than 10 m.

**Figure 1:**
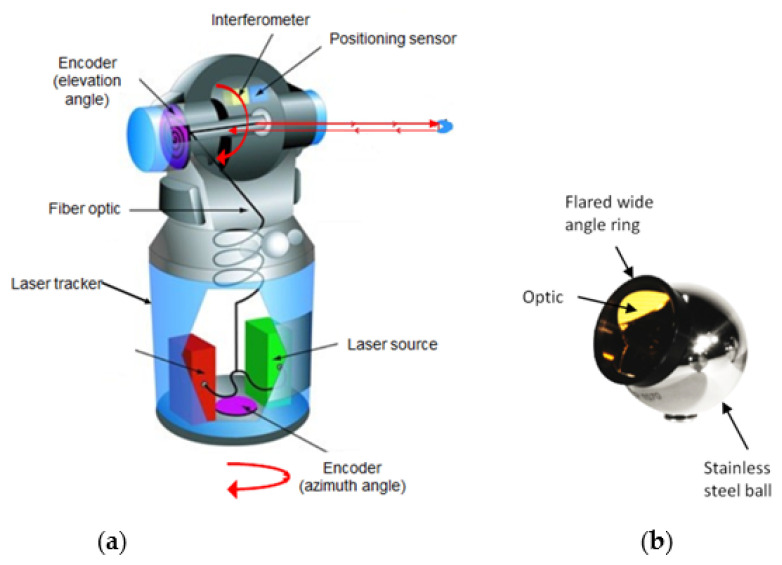
Laser tracker: (**a**) Laser tracker components; (**b**) SMR (Spherically Mounted Reflector).

An LT uses a laser interferometer to measure relative distances and optical encoders to measure two angles, *θ* (azimuth) and *φ* (elevation) [14] (see Figure 1).

LT specifications:

- Angular accuracy about 3.5 µm/meter;
- Angular resolution of ±0.018 arc seconds;
- System resolution about 0.1 µm;
- Infrared laser ADM accuracy better than ± 15µm.

#### 2.1.2. Coordinate Measuring Machines

A coordinate measuring machine (CMM) is a device that allows us to measure the geometry of different pieces by sensing points of the object with a probe. The probe displacement is measured in each of the three linear axes.

CMM specifications:

- CMM model: PMC 850 (Zeiss, Jena, Germania)
- Measurement accuracy of [2.3 + (L/300)] μm.
- Resolution of 0.1 µm.

### 2.2. Study of the Influence of the Laser Beam Incidence Angle on the Reflector

Reflector errors also have influence on the global error of the measurement system. The characterization of these components will allow us to study their influence in the measurement uncertainty.

The reflectors used in the measurement procedure were CCRs (Corner Cube Retroreflectors). They consist of three flat mirrors placed at a 90° angle to each other. The laser beam is reflected by the mirrors and returns in the same entry direction. Nevertheless, angular errors between mirrors and lack of uniformity of its coatings produce path errors in the laser beam. The spherical mounted retroreflector (SMR) consists of a CCR mounted inside a sphere. This type of reflectors is widely used in measurements with LTs. One of their characteristics is that they are static, so to measure a desired geometry we need to reposition the SMR for each measure or use multiple SMRs. The measured point is at the theoretical center of the sphere. The sphere diameter must be introduced in the measurement software to measure surfaces, since the point of the measured surface is at a distance of the measured point given by the sphere radius of the reflector. The sphere geometric center and the CCR optical center are ideally coincident. However, there is a distance between both centers in real SMRs that contributes to the SMR error. The roundness error of the sphere is another SMR error source. These errors, which produce uncertainty in LT distance measurements, can be caused by different factors in the manufacturing process. Geometric and optical errors that can be found in SMRs are described below.

Geometric errors can be:

- CCR geometric error. This error is caused by dihedral angle errors between each pair of reflective surfaces;
- CCR vertex position error. This error is a deviation between the real and the ideal CCR vertices.
- Optical errors are produced by:
- Incidence angle of the beam path;
- Errors in surface finish;
- Non-uniform coating.

**Figure 2:**
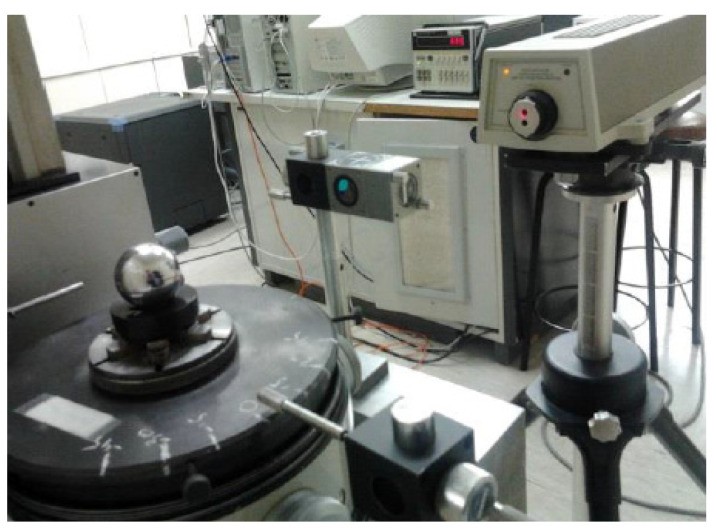
Roundness table SMR test.

These errors always affect the laser beam distance measurement, thus contributing to the LT measurement error. A test that determines the measurement error as a function of the incidence angle of the laser beam on the reflector was carried out to calculate them. To do this, a reflector with a magnetic base was mounted on the table of a high accuracy rounding machine (0.1 μm accuracy). The roundness table could be moved horizontally to center the piece placed on it. The piece surface displacement was recorded by means of a probe when the piece turned around the table center. Once the reflector was centered on the roundness table, the measurement was performed with an interferometer (IFM) that detected the position variations when the reflector rotated (see Figure 2).

### 2.3. LT Calibration

The calibration procedure allows us to identify the kinematic parameter errors in order to correct the measurement results. This procedure minimizes an objective function, formulated in terms of a non-linear least square problem [28]. The calibration method can be developed in five steps: determination of the kinematic model, data acquisition, geometric parameter identification, model evaluation and, finally, identification of the error sources and implementation of correction models [20].

#### 2.3.1. LT Kinematic Model

As is known, the kinematic model of an LT can be obtained using non-linear equations by expressing the joints with four parameters (*d_i_*, *a_i_*, *θ_i_* and *α_i_*), where:

- *d_i_* and *a_i_* correspond to the lengths of the links between successive reference systems
- *θ_i_* and *α_i_* are rotation angles of one system with respect to the other.

This method relates *i* frame to the (*i* − 1) frame by establishing four successive transformations [21], obtaining the homogenous matrix given by Equation (1).

(1) Ai−1i=Tz,d⋅Rz,θ⋅Tx,a⋅Rx,α=[cosθi−cosαi×sinθisinαi×sinθiai×cosθisinθicosαi×cosθi−sinαi×cosθiai×sinθi0sinαicosαidi0001]

**Figure 3:**
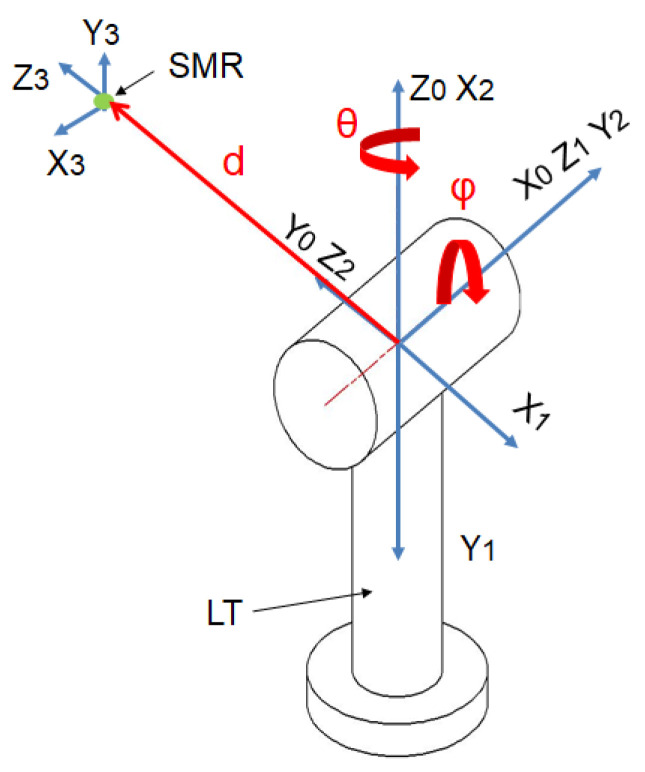
LT reference systems.

The LT uses two rotary joints and a linear axis as shown in Figure 3. The initial D–H parameters are represented in Table 1.

Rotation axis errors can be obtained by considering matrix *E_Rot_* (see Equation (2)).

(2) $$E_{Rot}=\left[\begin{array}{cccc} \cos\varepsilon R_{Y}\cdot \cos\theta R_{Z} & -\cos\varepsilon R_{Y}\cdot \sin\theta R_{Z} & \sin\varepsilon R_{Y} & \delta R_{X}\\ \cos\varepsilon R_{X}\cdot \sin\theta R_{Z\;}+\sin\varepsilon R_{X}\cdot \sin\varepsilon R_{Y}\cdot \cos\theta R_{Z} & \cos\varepsilon R_{X}\cdot \cos\theta R_{Z}-\sin\varepsilon R_{X}\cdot \sin\varepsilon R_{Y}\cdot \sin\theta R_{Z} & -\sin\varepsilon R_{X}\cdot \cos\varepsilon R_{Y} & \delta R_{Y}\\ \sin\varepsilon R_{X}\cdot \sin\theta R_{Z}-\cos\varepsilon R_{X}\cdot \sin\varepsilon R_{Y}\cdot \cos\theta R_{Z} & \sin\varepsilon R_{X}\cdot \cos\theta R_{Z}+\cos\varepsilon R_{X}\cdot \sin\varepsilon R_{Y}\cdot \sin\theta R_{Z} & \cos\varepsilon R_{X}\cdot \cos\varepsilon R_{Y} & \delta R_{Z}\\ 0 & 0 & 0 & 1 \end{array}\right]$$

where

- *δR_X_*, *δR_Y_* are radial errors;
- *δR_Z_* is an axial error;
- *εR_X_*, *εR_Y_* are tilt errors.

These error parameters vary depending on the type of joint. They are function of the rotation angle and have a periodic behaviour. Equations (3) and (4) show the modelling of these errors for an angle β, given by azimuth and elevation angles: β = {*θ,φ*}. In these equations, the first term is the encoder offset. The second harmonic presents good results. Performed simulations showed that by adding more harmonics, LT accuracy improvement is negligible.

(3) $${\varepsilon R}_{i}\left(\beta \right)={\varepsilon R}_{i1}+{\varepsilon R}_{i2}\times \sin\left(\beta +{R\varepsilon }_{i3}\right)+{\varepsilon R}_{i4}\times \sin\left(2\times \beta +{\varepsilon R}_{i5}\right)$$

(4) $${\delta R}_{i}\left(\beta \right)={\delta R}_{i1}+{\delta R}_{i2}\times \sin\left(\beta +{\delta R}_{i3}\right)+{\delta R}_{i4}\times \sin\left(2\times \beta +{\delta R}_{i5}\right)$$

Linear axis errors can be modelled as expressed by equation *E_Trans_* (see Equation (5)).

(5) $$E_{Trans}=\left[\begin{array}{c} 1\;-\varepsilon T_{Z}\;\;\;\;\;\varepsilon T_{Y}\;\;\;\;\;\delta T_{X}\\ \varepsilon T_{Z}\;\;\;\;\;1\;\;\;\;-\varepsilon T_{X}\;\;\;\;\delta T_{Y}\\ -\varepsilon T_{Y}\;\;\;\;\varepsilon T_{X}\;\;\;\;\;1\;\;\;\;\delta T_{Z}\\ 0\;\;\;\;\;\;\;\;\;0\;\;\;\;\;\;\;\;\;\;\;0\;\;\;\;\;\;\;1 \end{array}\right]$$

where

- *δT_X_*, *δT_Y_*, Δ*T_Z_* are linear errors;
- *εT_X_*, *εT_Y_*, *εT_Z_* are angular errors.

To modelling linear joint errors, a second order polynomial can be considered, as shown in Equations (6) and (7).

(6) $${\varepsilon T}_{i}={\varepsilon T}_{i1}+{\varepsilon T}_{i2}\times d+{\varepsilon T}_{i3}\times d^{2}$$

(7) $${\delta T}_{i}={\delta T}_{i1}+{\delta T}_{i2}\times d+{\delta T}_{i3}\times d^{2}$$

Finally, the LT kinematic model is given by multiplying the homogenous matrices obtained as shown in Equation (8).

(8) T03=A01×E0Rot1×A12×E1Rot2×A23×E2Trans3

#### 2.3.2. Data Capture Setup

To define the more suitable measurement scenario, some considerations should be taken into account in order to obtain a calibration procedure that is able to correct measurement errors in any position.

A sensitivity analysis can set up directions for data acquisition phase in which points are more sensitive to errors. This analysis, performed by simulation in a previous work, showed the error parameter dependence on distances and angles. Moreover, it predicted that positions given by minimum, zero, and maximum vertical angle are the ones that present more sensitivity [29]. These results were taken into account to design a test in real conditions. For this purpose, a large volume of approximately 6 × 6 × 6 m was arranged. Reflectors were located considering the sensitivity analysis. In this scenario, there were parameters that affected the equipment error in the same way in the entire range. On the contrary, some parameters presented a direct relationship with distance, and a periodic one with respect to azimuth angle. Moreover, the relationship was periodic with respect to azimuth angle, repeating every quarter turn of the vertical axis.

The study did not show a clear relation between elevation angle and errors.

Some error values were directly related with elevation angles. By contrast, for other parameters, the relationship was reverse.

These results imply that the following conditions must be considered to plan the calibration tests:

- Reflector positions should consider the maximum range of possible distances;
- The LT vertical axis should perform a minimum angle of π/2;
- Reflectors should be placed in positions that are possible to reach both in extreme positions and at the rotation equator of the elevation axis.

**Figure 4:**
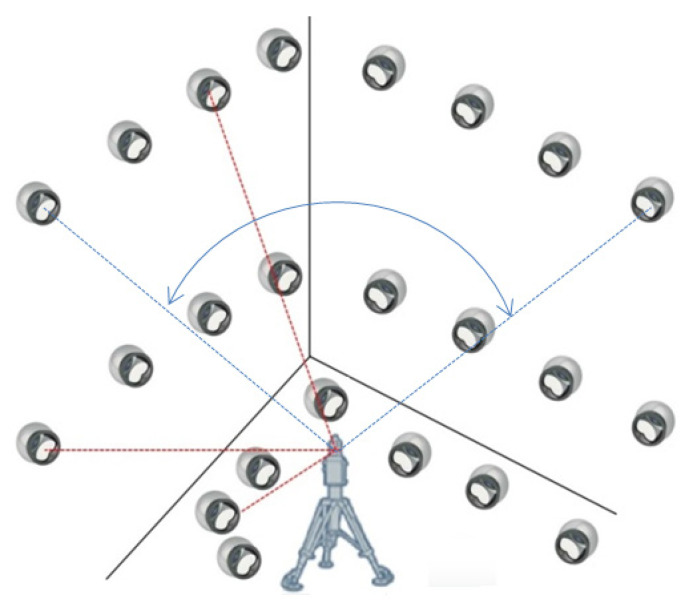
Reflector mesh positioning.

Following these three premises, the distribution of the reflectors was carried out as described. First, a diaphanous corner was arranged. The initial position of the LT was located in the bisector of the angle formed between two side walls, at a distance from the vertex of 4.25 m, obtaining an identical view of the two walls. Second, four additional LT locations were defined in different positions and orientations on the workshop floor (see Figure 4). Subsequently, the reflector positions were determined. They were placed on three levels of height:

- A first level of 8 reflectors at the maximum possible height, close to the ceiling, at 6 m high, thus obtaining the maximum elevation angle;
- A second level of 8 reflectors at the LT head height, approximately 2 m, which provides the neutral elevation angle;
- A third level of 8 reflectors in the ground level for the lower values of the angle.

**Figure 5:**
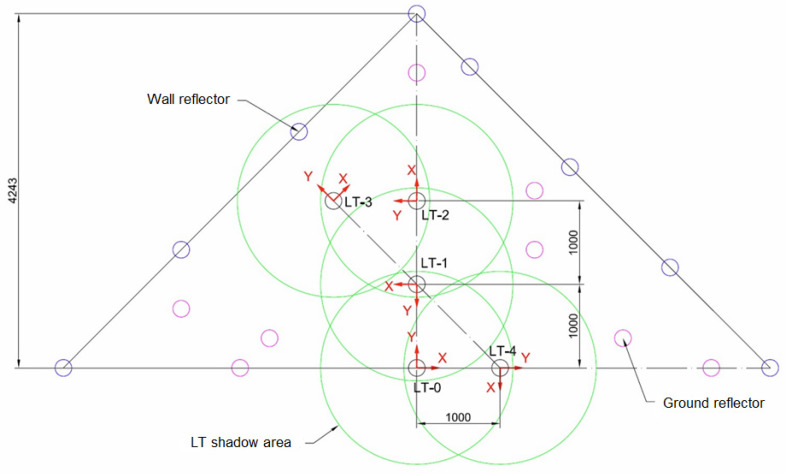
LT positions and shadow area projections of each LT.

Owing to the LT has a minimum elevation angle of −55°, there is a shadow area produced by each of the LTs. This shadow was represented by a cone with vertex at the origin of the laser with 110° opening. The shadow area projections of all the LT positions on the ground were drawn in Figure 5 in order to avoid placing any reflector in these areas. All reflectors should be measured from the 5 LT positions. The angle spanned from the initial LT position was 180°. Therefore, this range was the minimum azimuth rotation range spanned from all measurement positions.

Due to the reflector mesh nominal data are unknown, a reflector gauge was designed and measured by a CMM to have nominal data in the calibration procedure.

To determine the more suitable gauge in terms of accuracy and efficiency, different calibration strategies based on network measurements were analyzed by simulation in [24], using reflector gauges as nominal data. The minimum data gauge to get effective results, facing a greater range calibration, was a four-reflector gauge, adding the distances between reflectors belonging to the gauge in order to have redundant data in the optimization procedure. Tests performed showed that by adding more reflectors in the gauge, the improvement in the LT accuracy was negligible. Besides, a smaller gauge required less computational resources.

**Figure 6:**
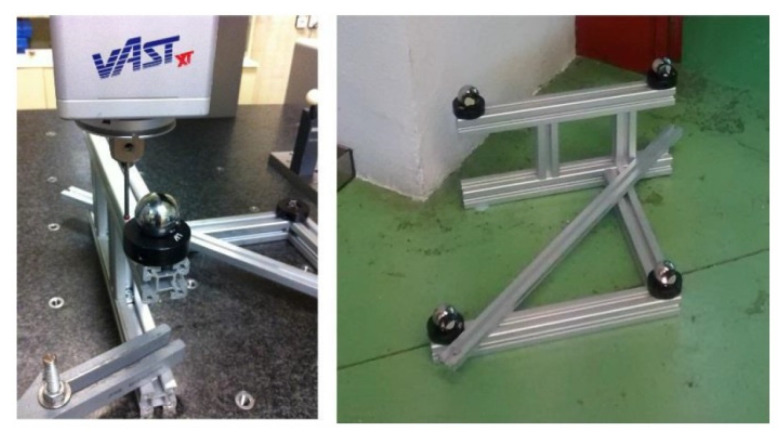
Reflector standard gauge.

These results were used to define the measurement scenario. Four reflectors were mounted on a rigid aluminum structure. The reflector supporting structure was built in a squad shape to prevent their positions to be on a plane parallel to one of the LT reference system (see Figure 6).

Their relative positions were then characterized by means of the CMM. The values obtained are shown in Table 2. These gauge reflectors were numbered from 25 to 28 in the calibration process.

This standard gauge was placed on the ground level near the corner.

**Figure 7:**
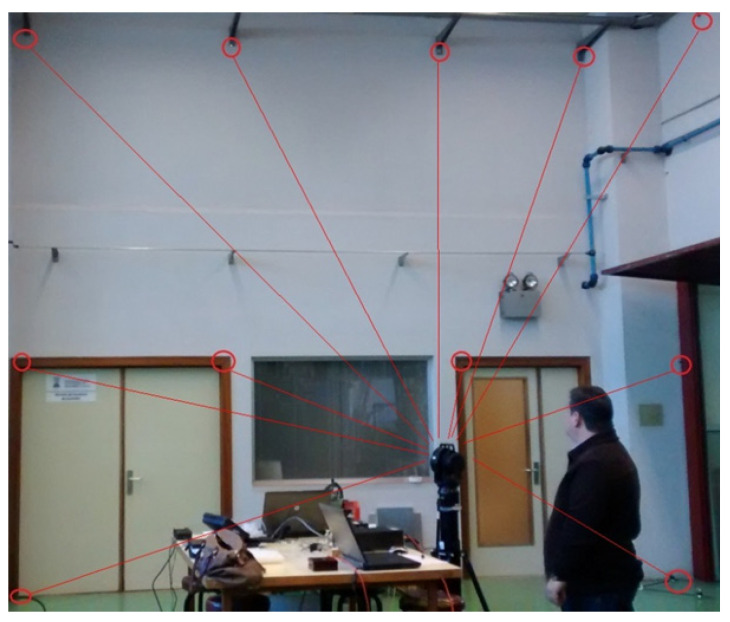
Reflector positions of the calibration procedure.

Thus, a total of 28 reflectors were measured from each of the 5 LT positions. Figure 7 shows the measurement scenario.

The steps carried out in this acquisition data phase were:

- To define the more suitable measurement scenario using a reflector mesh based on the results from a sensitivity analysis, thus considering those positions more sensitive to errors;
- To design the minimum data gauge to get effective results in terms of accuracy and efficiency;
- To measure the reflector gauge by using a CMM in the laboratory to obtain nominal data;
- To measure 28 reflectors (24 mesh reflectors and 4 gauge reflectors) by using an LT placed in 5 positions.

#### 2.3.3. Geometric Parameter Identification

Once the reflectors were measured from all the LT positions, the kinematic parameters that provide the optimum value of an objective function were identified. This function was formulated in terms of a non-linear least-square problem and it searches for the optimum values of the error parameters. It was defined as the sum of the difference of distances between every pair of reflectors of the mesh measured from every LT position.

The lack of nominal data in the data capture phase can present some problems in the geometric parameter identification results. In a real working scenario, the nominal positions of the reflector mesh are unknown. The use of only these measurements in the optimization process can provide some wrong results affected by rotation, deformation or a scale factor on the reflector mesh. To avoid this limitation, some known values were introduced in the objective function. The four-reflector gauge values, measured by the CMM, were considered as nominal values.

Therefore, the objective function to minimize for *n* reflectors and *m* LT locations is the sum of distance from point *k* to point *l*, measured from LT position *i* minus distance from point *k* to point *l* measured from LT position *j*. By adding the distances between reflectors belonging to the gauge in both, the first and the second term, and by considering gauges with *p* reflectors, Equation (9) was obtained:

(9) $$f=\overset{m-1}{\underset{i=1}{\sum }}\overset{m}{\underset{j=i+1}{\sum }}\overset{n-1}{\underset{k=1}{\sum }}\overset{n}{\underset{l=k+1}{\sum }}\left|d_{kl}^{i}-d_{kl}^{j}\right|+\overset{m}{\underset{i=1}{\sum }}\overset{n-1}{\underset{k=n-p+1}{\sum }}\overset{n}{\underset{l=k+1}{\sum }}\left|d_{kl}^{i}-d_{kl}^{CMM}\right|$$

where:

- $d_{kl}^{i}$ is the distance from reflector *k* to reflector *l* measured by the LT at location *i*;
- $d_{kl}^{j}$ is the distance from reflector *k* to reflector *l*, measured by the LT at position *j*;
- $d_{kl}^{CMM}$ is the distance from reflector *k* to reflector *l*, measured by the CMM, considered as nominal data.

In the calibration carried out, this general equation can be written as follows:

(10) $$f=\overset{4}{\underset{i=1}{\sum }}\overset{5}{\underset{j=i+1}{\sum }}\overset{27}{\underset{k=1}{\sum }}\overset{28}{\underset{l=k+1}{\sum }}\left|d_{kl}^{i}-d_{kl}^{j}\right|+\overset{5}{\underset{i=1}{\sum }}\overset{27}{\underset{k=25}{\sum }}\overset{28}{\underset{l=k+1}{\sum }}\left|d_{kl}^{i}-d_{kl}^{CMM}\right|$$

The phases of the calibration performed were:

- The optimization procedure was developed;
- The room temperature value was considered. Since the reflector gauge was measured in the laboratory in controlled conditions, the reflector gauge measurements were calculated in real conditions by considering the coefficient of thermal expansion of the gauge structure material and the temperature variation between the laboratory and the workshop;
- The error kinematic parameters were obtained by minimizing the difference between the distances measured of every pair of reflectors from all positions of the LT.

#### 2.3.4. Model Evaluation

The model evaluation step allows us to analyze if the optimization phase is able to reduce the dispersion of distance measurements.

The parameters obtained in previous section were used to correct the LT measurements. Measurements were collected in a working environment, so there were no reflector mesh nominal data available.

Two different validation criteria were used: a distance criterion and a coordinate criterion.

The distance criterion evaluated the difference in distance from every pair of SMRs, measured from LT position 1, as reference, and the other four LT positions before and after the calibration.

(11) $$\begin{array}{cc} Dif\text{\_}\;distance= & \overset{5}{\underset{m=2}{\sum }}\overset{27}{\underset{i=1}{\sum }}\overset{28}{\underset{j=i+1}{\sum }}(\sqrt{{\left(x_{i}^{1}-x_{j}^{1}\right)}^{2}+{\left(y_{i}^{1}-y_{j}^{1}\right)}^{2}+{\left(z_{i}^{1}-z_{j}^{1}\right)}^{2}}\\ & -\sqrt{{\left(x_{i}^{m}-x_{j}^{m}\right)}^{2}+{\left(y_{i}^{m}-y_{j}^{m}\right)}^{2}+{\left(z_{i}^{m}-z_{j}^{m}\right)}^{2}}) \end{array}$$

The coordinate criterion evaluated distances in coordinates for every SMR, measured from the 5 LT positions. To carry out this evaluation, the reference system of the SMR positions was changed to be all represented in the same reference system, *SR*_1_, using a least-square adjustment. This evaluation was carried out by the data acquisition software, setting the correspondence from at least three SMR positions of each LT position.

(12) $$Dif\text{\_}distance=\overset{4}{\underset{m=1}{\sum }}\overset{5}{\underset{n=m+1}{\sum }}\overset{28}{\underset{i=1}{\sum }}\left(\sqrt{{\left(x_{i\text{\_}SR_{1}}^{m}-x_{i\text{\_}SR_{1}}^{n}\right)}^{2}+{\left(y_{i\text{\_}SR_{1}}^{m}-y_{i\text{\_}SR_{1}}^{n}\right)}^{2}+{\left(z_{i\text{\_}SR_{1}}^{m}-z_{i\text{\_}SR_{1}}^{n}\right)}^{2}}\right)$$

#### 2.3.5. Correction Model Implementation

The verification step will allow us to know if the calibration is really correcting the LT measurements.

Parameters were calculated at 27 °C. They were then verified with nominal values measured using the CMM at 20 °C.

In this phase, data were obtained in the laboratory. A set of 17 reflectors located on the table of a CMM were measured. The error parameter values calculated in the calibration procedure, in a real working environment, were then used to verify the calibration behavior by correcting laboratory measurements.

The steps performed in this phase were:

- A set of reflectors were located on the table of a CMM;
- The reflectors were measured with both the CMM and the LT from different positions in the laboratory;
- The measurements obtained by the CMM were considered as nominal values;.
- The measurements obtained by the LT in the laboratory were corrected with the geometric parameters obtained in the calibration phase in the working environment;
- Both measured and corrected LT measurements were expressed in the CMM reference system using homogeneous transformation matrices;
- It was evaluated whether the corrected LT measurements were closer to the CMM measurements than the uncorrected LT measurements.

(13) $$\begin{array}{cc} Err= & \overset{4}{\underset{m=1}{\sum }}\overset{5}{\underset{n=m+1}{\sum }}\overset{16}{\underset{i=1}{\sum }}\overset{17}{\underset{j=k+1}{\sum }}(\sqrt{{\left(x_{i}^{m}-x_{j}^{CMM}\right)}^{2}+{\left(y_{i}^{m}-y_{j}^{CMM}\right)}^{2}+{\left(z_{i}^{m}-z_{j}^{CMM}\right)}^{2}}\\ & -\sqrt{{\left(x_{i}^{n}-x_{j}^{CMM}\right)}^{2}+{\left(y_{i}^{n}-y_{j}^{CMM}\right)}^{2}+{\left(z_{i}^{n}-z_{j}^{CMM}\right)}^{2}}) \end{array}$$

(14) $$Err=\overset{5}{\underset{m=1}{\sum }}\overset{17}{\underset{i=1}{\sum }}\left(\sqrt{{\left(x_{i\text{\_}SR_{1}}^{m}-x_{i\text{\_}SR_{1}}^{CMM}\right)}^{2}+{\left(y_{i\text{\_}SR_{1}}^{m}-y_{i\text{\_}SR_{1}}^{CMM}\right)}^{2}+{\left(z_{i\text{\_}SR_{1}}^{m}-z_{i\text{\_}SR_{1}}^{CMM}\right)}^{2}}\right)$$

Although the proposed method is effective in verifying the model and only requires a standard gauge, the availability of long length reference length instruments would allow us to cover a greater range and would be more effective.

## 3. Results

### 3.1. SMR Results

**Figure 8:**
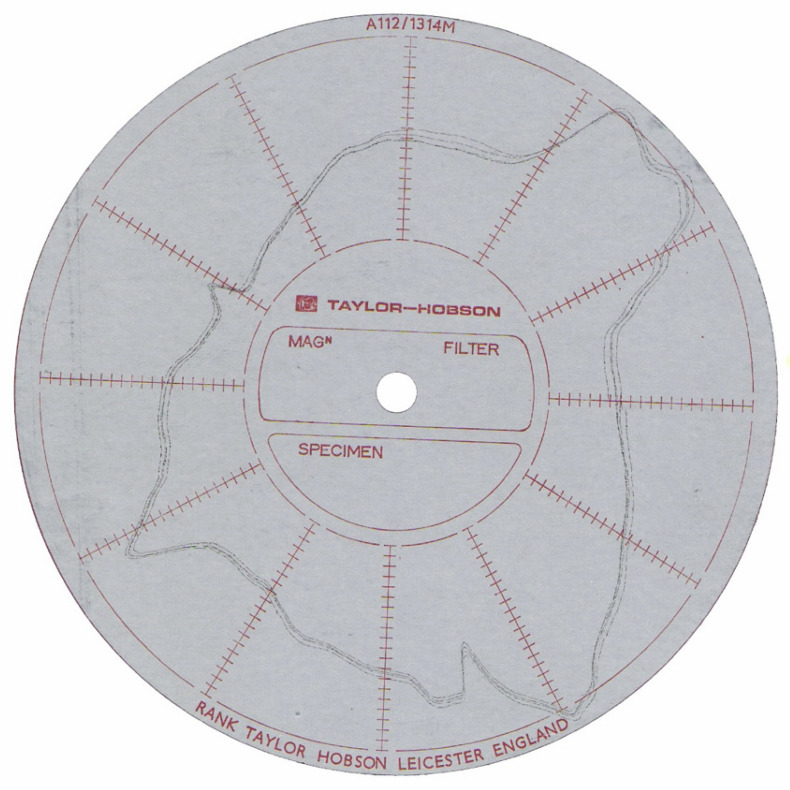
Centering error: eccentricity and roughness errors of the SMR sphere (2 μm scale).

As was detailed in Section 2.2., a SMR was mounted and centered on a roundness table. The piece surface displacement was recorded to characterize the eccentricity and roughness errors of the SMR sphere. The position variations were detected using an IFM when the reflector rotated, as shown in Figure 8. The range of measurable variation was 2 μm.

The incidence angle of the laser beam contributes to the measurement uncertainty. This uncertainty will mainly appear in the measured values.

The results showed that the incidence angle admissible in SMRs was close to ±30°. Rotations were then performed in 7.5° increments, thus covering the entire allowable range.

Since the reflector presents three symmetry planes located at 120°, it is not easy to relate results with the initial orientation of the symmetry planes. However, in the view of this test, it can be observed that there is a clear tendency to present higher errors in inclination angles having more negative values.

**Figure 9:**
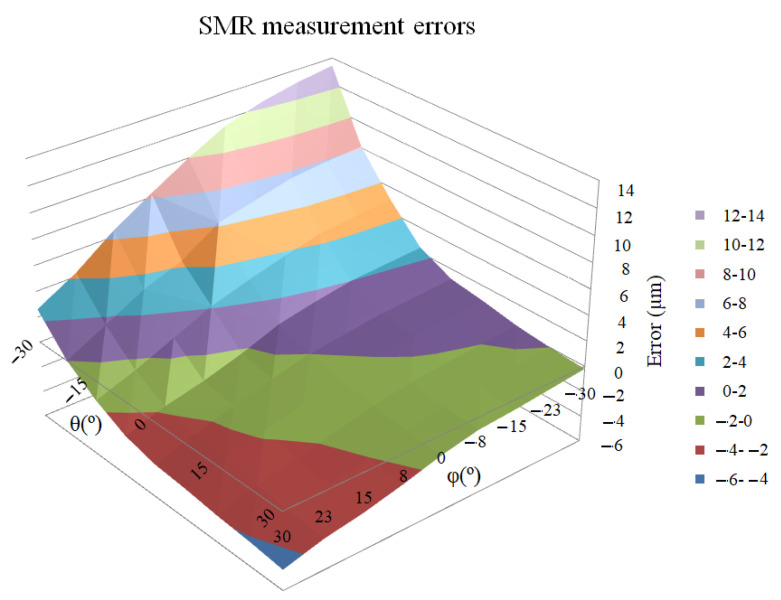
SMR measurement errors (µm) depending on the incidence angle (°).

Although the difference is not significant for small angles, when approaching the limit incidence angles, very significant errors can be found, up to 13 µm, which provide a very important contribution to the LT measurement error (see Figure 9). However, these values only appear in extreme angles. The mean error at central angles is around 3 µm.

The contribution of the angle of incidence of the laser beam in the measurement uncertainty will appear mainly in the value of the measured distance. It should be negligible in the angular measurements.

### 3.2. Calibration Results

This section presents results obtained in the calibration procedure performed, following the phases explained in Section 2.3.

#### 3.2.1. Calibration Evaluation Results

Once calibration parameters were determined by minimizing the objective function presented in Equation (9), two validation criteria were used to obtain how the average distance difference between every pair of reflectors is reduced.

Distance criterion:

- The distance between every pair of reflectors from every LT position was calculated;
- The average of the distances calculated was obtained. The distance difference is obtained as the absolute distance of the difference between the distance measured from a position and the average;
- The average distance difference of every pair of reflectors is the average of the distance difference.

**Figure 10:**
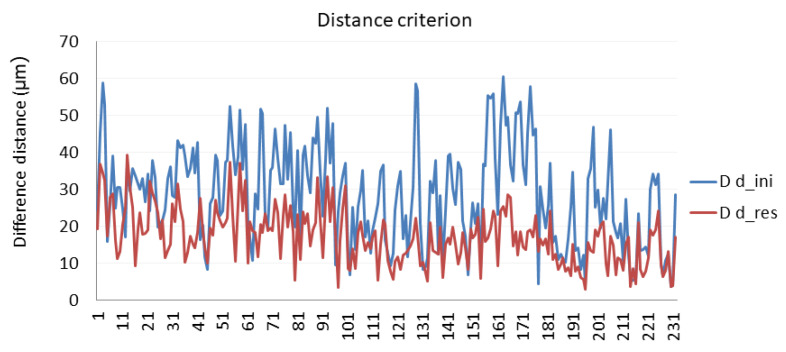
Calibration evaluation according to the distance criterion.

Figure 10 shows the average distance difference for the distance criterion. This figure compares the initial values (*D d_ini*) and the corrected ones (*D d_res*).

Coordinate criterion

**Figure 11:**
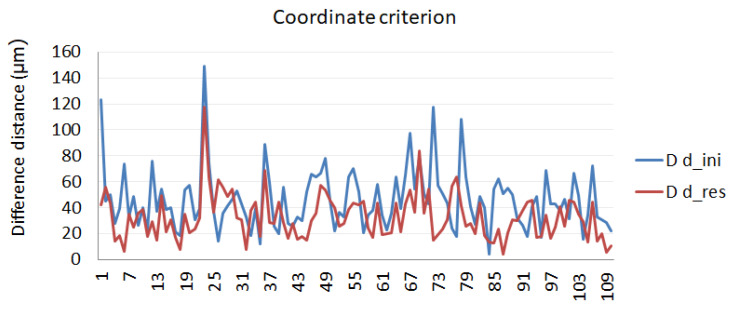
Calibration evaluation according to the coordinate criterion.

Reflector measurements are all calculated in the same reference system by means of a least squares fit. The position of every reflector measured from 5 positions of the LT is compared with the position of the same reflector measured from the other LT positions to obtain the Euclidean distance. Figure 11 presents the results obtained in the calibration procedure following the coordinate criterion.

Table 3 shows the performance model for both distance and coordinate criteria. The influence that the calibration criterion has on the calibration result can be noted.

As can be seen from a comparison of the two criteria, the distance criterion showed better performance than the coordinate criterion, and fitted better the optimization procedure. Moreover, this last criterion provided results with more difference between the maximum and the minimum, offering a wider range.

#### 3.2.2. Verification of the Calibration Procedure Developed

The analysis of the LT accuracy improvement, shown in Table 3, is based on indirect evaluation methods since it is not possible to compare measured and corrected data values with nominal values. Reflectors were placed in pre-set positions but without any accuracy, simulating a real working environment. Moreover, no measuring instrument which can give us the real reflector positions was available with better accuracy than the LT.

To verify the calibration procedure, the LT behavior was evaluated with the set of the identified parameters obtained in the geometric parameter identification phase, in other positions different from those used in the optimization process. Nominal data were obtained in the laboratory. A set of reflectors located on the table of a CMM were measured by the LT and by the CMM. The error parameter values calculated in the calibration procedure, in a real working environment, were then used to verify the calibration behavior.

Furthermore, the test carried out allowed us to evaluate if the calculated error parameters were affected by the temperature difference between the workshop measurement site and the metrological laboratory.

The reflector mesh was measured in the workshop at 27 °C by the LT.

A standard gauge was measured in the laboratory using a CMM at 20 °C.

In order to consider the standard gauge measurements as nominal values in the LT calibration procedure, temperature variation and linear-expansion coefficient of the standard gauge were taking into account. Equation (15) reflects this correction.

(15) $$d_{workshop}=d_{lab}^{}+\left(T_{workshop}-T_{lab}\right)\times \lambda$$

where

- *d_wor__kshop_*: distances obtained measuring in the workshop at 27 °C;
- *d_lab_*: distances obtained measuring in the laboratory at 20 °C;
- *T_workshop_*: Temperature in the workshop (27 °C);
- *T_lab_*: Temperature in the laboratory (20 °C);
- *λ*: Linear-expansion coefficient of the material.

**Figure 12:**
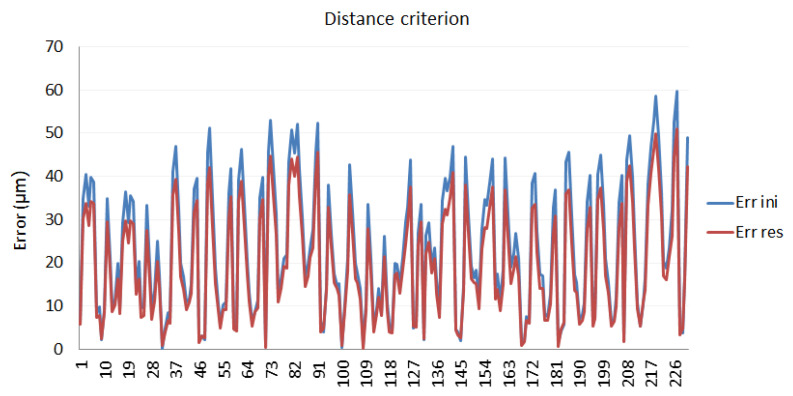
Calibration verification using the distance criterion.

**Figure 13:**
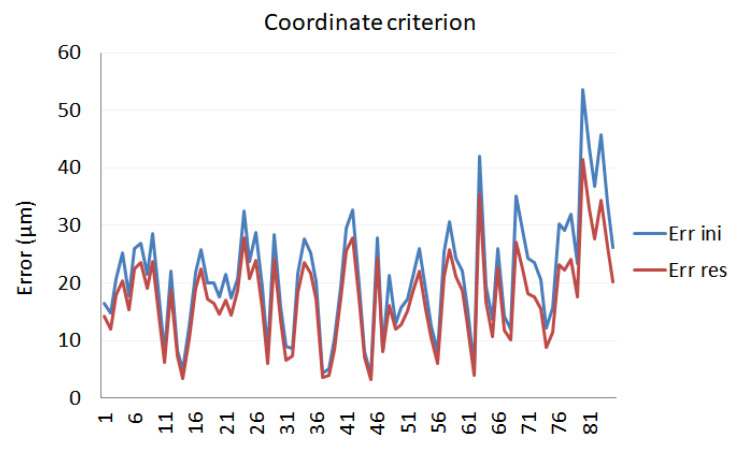
Calibration verification using the coordinate criterion.

The results obtained in the verification phase are shown for distance and coordinate criteria in Figure 12 and Figure 13, respectively.

The improvement values achieved with the calibration, applied to laboratory measurements, are given by Table 4.

As can be seen from a comparison of the verification with both criteria, the results do indicate that these improvements are quite similar, although the distance criterion improvement is slightly higher.

## 4. Discussion

A new calibration procedure that improves the accuracy of an LT was performed and validated. The method is based on network measurements and was carried out in a real working environment.

The different calibration phases were presented. The main contributions of the paper are:

- The more suitable measurement scenario was designed, according to the results of a sensitivity analysis test. A set of reflectors were located in those points more sensitive to errors. This design defined those reflector positions that provided the maximum range of possible distances. The LT vertical axis moved a minimum angle of π/2. The reflector positions included those points in which the LT was working in extreme positions and at the rotation equator of the elevation axis;
- The SMR analysis showed that although there are no symmetries depending on the angle and the error is around 3 µm in central angles, remarkable trends towards incidence angles can be noted that significantly increase the error, up to 13 µm in extreme angles;
- The kinematic parameters obtained in the optimization phase were used to correct the LT measurements of a reflector mesh collected in a working environment. Distances between reflectors belonging to the mesh, measured from different LT positions, were considered in the optimization process. The distance values between a four-reflector gauge were measured by the CMM and considered as nominal values in the calibration to avoid that results could be affected by a rotation, deformation, or a scale factor on the reflector mesh, since no reflector mesh nominal data were available. The two criteria used improved LT accuracy. However, the findings of the current study revealed that the distance criterion fitted better the optimization procedure and provided wider range than the coordinate criterion. The LT accuracy improvement with distance criterion was considerably higher than with coordinate criteria (40.1% versus 14.5% respectively);
- To verify LT calibration results, nominal data of a new reflector mesh were obtained in the laboratory, using a CMM and an LT placed in different positions from those utilized in the optimization process. The error parameter values obtained before in the calibration in the workshop were then used to correct these new measurements. In this case, both distance and coordinate criteria showed a quite similar LT accuracy improvement (18% versus 25.5%, respectively). These results confirm that the calibration is valid for any measurement scenario, allowing the end-user to improve the LT accuracy around 25.5%.

One of the most important advantages of the procedure developed is that it will allow an LT user to know if the LT works properly after a while in a working environment without requiring specialized equipment, in a fast and easy way.

This calibration can be performed in those LT constructions having the beam source in the rotating head. Nevertheless, the procedure could be adapted to an LT having the beam steering mirror by developing its kinematic model.

## Figures and Tables

**Table 1:** D–H initial parameters.

*i*	a_*i*_ (mm)	α_*i*_ (°)	d_*i*_ (mm)	θ_*i*_ (°)
1	0	−90	0	θ − 90
2	0	90	0	φ − 90
3	0	0	d	−90

**Table 2:** Distance values between gauge reflectors.

		Source Reflector		
		25	26	27
Target reflector	26	357.6066	590.1060	355.9437
	27	388.0144	386.7815	
	28	454.4203		

**Table 3:** Model behavior in the calibration procedure for different criteria.

		Coordinate Criterion	Distance Criterion
Initial Difference distance (µm)	Maximum	149	60
	Average	46	29
Residual Difference distance (µm)	Maximum	118	39
	Average	39	17
	Improvement (%)	14.47	40.1

**Table 4:** Verification values in the calibration procedure.

		Coordinate Criterion	Distance Criterion
Initial error (µm)	Maximum	54	84
	Average	21	24
Residual error (µm)	Maximum	41	53
	Average	17	18
	Improvement (%)	17.98	25.53

## Data Availability

Not applicable.
